# Clinical Remarks on Surgical Cases Occurring in the Buffalo Hospital of the Sisters of Charity

**Published:** 1870-12

**Authors:** Julius F. Miner


ART. V.—*Clinical Remarks on Surgical Cases occurring in the Buffalo Hospital of the Sisters of Charity*, *by* JULIUS F. MINER, M. D.

REPORTED BY W. W. MINER, MEMBER OF THE CLASS.

———

CASE VII.—*Amputation at Knee Joint.* The young patient, W—— M——, ten years of age, who is now brought before you, received a railroad injury on the thirteenth of August last, which necessitated the amputation of his leg near the junction of the middle and lower thirds of the tibia. Nine weeks have now elapsed since the operation. You notice that the integuments of the stump are swollen, and that pus is exuding from several openings in the cicatrix and from other points in the tissue of the leg above, while the general condition of the patient is that of great anæmia and depression.

It has been evident for some time that three or four inches of the lower portion of the tibia is diseased. This condition may be occasioned by injury above the point of amputation, by epiphyseal separation of the head of the bone from the shaft, or by the cutting off of sufficient nutritive supply to the bone. Whatever cause may have effected this pathological condition, the possibility of the removal of the diseased bone, by the natural process of absorption, is precluded. This point is determined by the general condition of the patient, and the dangers which exist from the absorption of pus. Our method of procedure is then plainly, that of removal of the diseased portion of bone by operative interference.

After inducing anæsthesia, and exposing the tibia to view, with the intention of exsecting such a part of it as was diseased, it was found that a deposit of new bone had partly encircled the diseased portion, and that so much of the tibia was involved as to necessitate amputation at the knee joint, which was then performed.

Amputation at the knee joint presents a wide field for remark, as well as it also does for experimental observation. Notwithstanding the fear that surgeons used to have of entering joints and exposing joint surfaces, modern experience has shown that such procedure is not necessarily followed by any untoward consequences. The history of the various o perations of exsection, confirm the belief that operations involving joints are not only safe, but are frequently advisable. The propr iety of amputations at the joints, is rapidly coming into recognition by surgeons generally.

Amputation at the knee joint is to be preferred to amputation of the leg made so near the joint as to involve the cancellous structure of the head of the tibia. There is much less danger from suppuration, in operating at the joint, than there is when the cancellous structure of a bone is divided by a saw, in which latter case the fragile bony texture is more or less injured, and its porous interspaces impacted with saw dust fragments of bone, whose removal must afterwards be effected by suppuration or absorption. Amputation at the joint will give a better stump than that at the tubercle of the tibia: it will have a broader extremity, and one whose integuments do not retract, and which is not, to any considerable extent, sensitive.

In making the operation I prefer to have the short flap anteriorly: the attachment of the patella to the tibia is severed, and the flap dissected up to the joint; the joint itself is then carefully separated, and the posterior flap made of sufficient size to well cover the broad articular surface. The patella may be left in the anterior flap and be drawn down upon the condyles of the femur, to whose irregularities it is so well adapted. Adhesive straps are used to support the integument of the stump, and between these are left intervals for the escape of pus, and for the subsequent convenient examination of the parts. A compress wet with carbolic acid water is usually applied as a dressing.

On the next morning after the operation our little patient rallied completely, although he was so near dead from the profuse discharge which existed prior to the operation, that stimulants were carefully and constantly administered him. The wound is now, (six weeks afterwards,) entirely closed, and the patient so fleshy and healthy in appearance as to be hardly recognizable. This is the third, at least, of the cases of amputation at the knee joint that have come within my own experience, each of which have been unexceptionably successful.

Case VIII.—*Encephaloid Cancer.*—The present case, which is brought to your notice through the kindness of Dr. Cronyn, is one of encephaloid cancer, arising probably in the sub-maxillary gland, and affecting the tissues about the inferior maxillary bone. It has progressed to the condition of an open cancer; and, though its removal is not now proposed, yet it furnishes you an opportunity of observing the progress of carcinomatous disease. The varieties of cancerous growths may be classed under three general heads: hard, soft and black cancer. Under the term hard cancer is included scirrhus and epithelioma; under soft cancer, encephaloid and colloid; while black cancer is otherwise termed melanosis. While we do not propose to speak particularly, at present, concerning the causes and pathology of cancer, still I may say that, in practice, I have found that, if these growths are removed before they present the character of an open ulcer, their recurrence seems to be delayed, perhaps averted; whereas, if not removed early, the whole system of the person affected seems to be so implicated in the disease that operative interference is altogether useless. As the case is brought to your attention repeatedly, you notice that the area involved increases in size. Its proximity to the carotid artery suggests that that vessel may soon be involved; but it is found that the coats of arteries resist the progress of this disease, and remain intact considerably longer than do other tissues. The patient is to be relieved of unnecessary pain by the administration of opiates in such doses as are found requisite.

Case IX.—*Bubo.*—A bubo is an inflammatory enlargement of a lymphatic gland. It oftenest occurs in the glands of the inguinal region, and its occurrence may be due to the absorption of venereal poison, or of pus from a suppurating surface on the lower extremity. Generally, buboes have a venereal origin. The period at which syphilitic buboes appear is two weeks or more after the occurrence of the chancre. They are indolent in their progress, and are of two kinds, according as they succeed the indurated or the non-specific form of chancre. A bubo which succeeds a non-specific chancre or chancroid does generally suppurate, and the pus which is discharged is not capable of producing constitutional syphilis. Those buboes which succeed true syphilitic chancres do not so generally suppurate; but when they do, the pus which escapes from them will produce the constitutional disease. Where they have progressed to near suppuration before your attention is called to them, as is generally the case, I do not believe there is any method of preventing their progress. Suppuration and discharge of pus is the common and natural termination, and if you can assist this natural process, you will be hastening the processes of recovery. Various means of treatment are recommended, such as quiet, pressure, stimulating applications of iodine, mercury, etc. Before fluctuation is apparent, poultices are to be applied, and afterwards they can be opened as early as you are able to detect in them a collection of pus.

CASE X.—*Enucleation of the Eye-ball.*—The young patient now introduced received an injury to his left eye-ball some four months since, by which vision in that organ was speedily lost. The preservation of the injured eye is no longer of importance, and the only point to which attention is needed is as to the safety of the other eye, and the importance of careful attention to this point, in such cases can scarcely be exaggerated. The inflammation which now exists in the remains of the left eye, and which is liable to recurrence at any future period after the perfect healing of the parts may have been effected, constitutes a constant source of anxiety and watchfulness. We have constantly to be in fear of the incitement of sympathetic inflammation in the other eye, the only organ of vision left to the patient. It is a point of which we are to be aware, that an inflammatory condition is sometimes transmitted from one part to another by means of nervous connection; and this kind of sympathetic inflammation, called sympathetic ophthalmia, is a notable instance of such transmission. The uninterrupted progress of sympathetic ophthalmia is fatal to the organ in which it occurs. If it is found that intolerance of light or photophobia is beginning to be felt in the hitherto uninjured eye, if there is dimness of vision or lachrymation, we are to be on the alert for such a condition no medicine in the world will influence or retard. Enucleation, or removal of the remains of the injured globe, should be performed without delay, when it is certain that the other eye is suffering in any great degree from its remaining. The changes which are produced by sympathetic ophthalmia are in the delicate nervous tissue of the retina, and such change is not accompanied by very demonstrative symptoms. The instances in which physicians have unnecessarily and unknowingly allowed this disease to produce irremedial blindness are not few or infrequent. In making the operation it is usual to produce anæsthesia, and then having seized a portion of the conjunctival membrane with a pair of forceps, to make with the scissors an opening of sufficient size to admit a pair of curved scissors made for this purpose, holding the eye with the forceps, you may divide with these scissors the conjunctival and muscular attachments of the eye, and at length sever the optic nerve near its entrance to the eye. It is essential, if enucleation be not performed, that at least the whole of the iris and its ciliary attatchment be removed, as the ciliary region is believed to be the seat of sympathetic inflammation. In this manner the cause of all danger of sympathetic ophthalmia will at once be removed. After the parts have healed, and before they have greatly contracted, an artificial eye may be inserted.

